# Long-Term Cardiovascular Toxicity of Immunotherapy: Too Important to Ignore

**DOI:** 10.3390/ijms26189100

**Published:** 2025-09-18

**Authors:** Andrea Camerini, Alessandro Inno, Maria Laura Canale, Stefano Oliva, Andrea Tedeschi, Alessandra Greco, Marzia De Biasio, Nicola Maurea, Irma Bisceglia, Luigi Tarantini, Giuseppina Gallucci, Carmine Riccio, Giovanna Geraci, Claudio Bilato, Alessandro Navazio, Attilio Iacovoni, Furio Colivicchi, Massimo Grimaldi, Fabrizio Oliva

**Affiliations:** 1 Medical Oncology, Versilia Hospital, Azienda USL Toscana Nord-Ovest, 55041 Lido di Camaiore, Italy; andrea.camerini@uslnordovest.toscana.it; 2 Medical Oncology, IRCCS Ospedale Sacro Cuore Don Calabria, 37024 Negrar di Valpolicella, Italy; alessandro.inno@gmail.com; 3 Cardiology, Versilia Hospital, Azienda USL Toscana Nord-Ovest, 55041 Lido di Camaiore, Italy; 4 UOSD Cardiologia di Interesse Oncologico, IRCCS Istituto Tumori “Giovanni Paolo II”, 70124 Bari, Italy; s.oliva@oncologico.bari.it; 5 Cardiology, “Guglielmo da Saliceto” Hospital, 29121 Piacenza, Italy; andrea.tedeschimd@gmail.com; 6 Cardiology Division, Fondazione IRCCS Policlinico San Matteo, 27100 Pavia, Italy; a.greco@smatteo.pv.it; 7 Cardiology, Azienda Sanitaria Universitaria Friuli Centrale, 33100 Udine, Italy; marzia.debiasio@asufc.sanita.fvg.it; 8 Cardiology Division, Istituto Nazionale Tumori, IRCCS Fondazione G. Pascale, 80131 Napoli, Italy; segreteria3profmaurea@gmail.com; 9 Servizi Cardiologici Integrati, Dipartimento di Scienze Cardio-Toraco-Vascolari, Azienda Ospedaliera San Camillo Forlanini, 00152 Roma, Italy; irmabisceglia@gmail.com; 10 Cardiology, Arcispedale Santa Maria Nuova Hospital, Azienda USL di Reggio Emilia-IRCCS, 42122 Reggio Emilia, Italy; luigi.tarantini@gmail.com (L.T.); alessandro.navazio@ausl.re.it (A.N.); 11 Private Practice, 85025 Melfi, Italy; pina.gallucci@tiscali.it; 12 Dipartimento Cardio-Vascolare, AORN Sant’Anna e San Sebastiano, 81100 Caserta, Italy; carminericcio8@gmail.com; 13 Cardiology, Sant’Antonio Abate Hospital, ASP Trapani, 91016 Erice, Italy; giovannageraci68@icloud.com; 14 Cardiology, Ospedali dell’Ovest Vicentino, Azienda ULSS 8 Berica, 36100 Vicenza, Italy; claudio.bilato@aulss8.veneto.it; 15 SSD Chirurgia dei Trapianti e del Trattamento Chirurgico dello Scompenso, Dipartimento Cardiovascolare, ASST Papa Giovanni XXIII, 24127 Bergamo, Italy; aiacovoni@asst-pg23.it; 16 UOC Cardiologia Clinica e Riabilitativa, Presidio Ospedaliero San Filippo Neri-ASL Roma 1, 00135 Roma, Italy; furio.colivicchi@gmail.com; 17 UOC Cardiologia-UTIC, Ospedale Miulli, 70021 Acquaviva delle Fonti, Italy; massimo.grimaldi@cardio.uniba.it; 18 Cardiologia 1-Emodinamica, Dipartimento Cardio-toraco-vascolare “A. De Gasperis”, ASST Grande Ospedale Metropolitano Niguarda, 20162 Milano, Italy; fabri.oliva@gmail.com

**Keywords:** immunotherapy, atherosclerosis, immune checkpoint inhibitors, cancer, cardiovascular toxicity, myocardial infarction, ischemic stroke

## Abstract

Immunotherapy improved survival in a significant number of cancer patients as never before. Its benefits spread across cancer types and stages, and new drugs, new combination, and new indications are on the way. Both atherosclerosis and cancer are associated with the same risk factors, molecular processes, and a persistent inflammatory state that is attributed to immune system dysregulation. Clinicians are now facing a new category of patients that are long survivors/cured by immunotherapy. While short-to-medium-term side effects of immunotherapy are well characterized, long-term exposure to immune checkpoint inhibitors could be associated with new and unpredicted toxicity with a potential impact on survival, reducing the clear advantage of immunotherapy. Because of this, it is clear that the worsening of atherosclerosis emerges as the most relevant long-term side effect, translating into an increased incidence of atherosclerosis-related cardiovascular events, such as myocardial infarction, ischemic stroke, and peripheral artery disease. We review the available evidence of this relevant association, providing an overview for all clinicians involved in the multidisciplinary cancer care process.

## 1. Introduction

For several solid and hematological tumors, across all stages, from both metastatic to early disease, immunotherapy (IT), based on monoclonal immune checkpoint inhibitor antibodies (ICIs), is the cornerstone of cancer treatment [1,2]. ICIs, by blocking inhibitory immune checkpoints, inhibit the T-lymphocyte inhibitory pathways and thus reactivate their anti-tumor cytotoxic response [3]. Different proteins on immune cell surfaces or in the tumor microenvironment are targeted by currently approved ICIs, such as cytotoxic T-lymphocite-associate-4 protein (CTLA-4), programmed cell death protein 1 (PD-1) and its ligand (PD-L1), lymphocyte-activation gene 3 protein (LAG-3), and T cell immunoglobulin and ITIM domain (TIGIT), but many other ICIs are under development [4]. Different ICIs act in different phases and sites of immune response, i.e., CTLA-4 is implicated in the priming phase within local/regional lymph nodes, while PD-1/PD-L1 works on the effector phase at tumor microenvironment level [5].

Positive results in terms of a cure in adjuvant/neo-adjuvant settings and long-term disease control for advanced stages are now countless [6,7,8,9], and IT (alone or in combination with target agents, chemotherapy and radiotherapy) is the gold standard for the treatment of many cancers with millions of patients exposed [10,11]. ICIs have a distinct toxicity profile as a result of their mechanism of action. In fact, they may lead to immune-related adverse events (irAEs), which can possibly affect any organ or system, including the cardiovascular system, by inducing a lack of self-tolerance [12,13]. Short-to-medium-term side effects of IT have been widely described [14] and various “acute/early” cardiovascular system-related side effects including myocarditis, pericarditis/pericardial effusion, arrhythmias, and vasculitis have been reported [15]. Given the positive effect of IT on patients’ survival with prolonged exposure to ICIs, recently late-onset (delayed) and long-term toxicities surfaced and gained the attention of clinicians [16,17]. Although the true frequency of late-onset and long-term irAEs is unknown, it is thought to be in the range of 5%. These events may occur months during treatment or even after treatment discontinuation and have been usually underreported, contributing to a significant clinical concern [18]. Growing evidence suggests that long-term toxicity of IT on the cardiovascular system may be represented by atherosclerotic-related events.

This review synthesizes existing mechanistic and clinical evidence on atherosclerosis in ICI-treated patients and translates it into clinical management guidance (risk stratification, monitoring, prevention). We do not advance a new mechanistic theory; rather, we align established immunobiology (e.g., PD-1/PD-L1, CTLA-4, LAG-3 pathways) with emerging clinical signals to identify knowledge gaps and priorities for prospective research.

## 2. Immune Checkpoint Inhibition Therapy and the Atherosclerotic Process

Atherosclerosis is a disease affecting the intima, characterized by the formation of plaques primarily composed of lipids and extracellular matrix proteins. It can remain asymptomatic for decades before manifesting clinically through various mechanisms, including progressive plaque enlargement that may reduce vascular flow, transition from stable to unstable plaques with subsequent rupture and thrombus formation, potentially resulting in vascular occlusion or embolism, dysregulated vascular tone promoting vasoconstriction under inappropriate conditions such as exercise, and, ultimately, aneurysm formation due to medial atrophy secondary to intimal atherosclerosis [19]. In cancer, these mechanisms may be further exacerbated by systemic inflammation and oncologic therapies [19].

It is now well established that atherosclerosis represents a chronic immune-inflammatory response to intimal injury. In this context, inhibitory immune checkpoints may help mitigate intimal inflammation, thereby exerting a protective effect against atherosclerosis. Several preclinical studies suggest that the CTLA-4, PD-1/PD-L1, and LAG-3 pathways play a protective role in atherogenesis, whereas their blockade may contribute to atherosclerosis progression [20]. Notably, most preclinical models indicate that ICI-related atherogenesis is predominantly mediated by CD4^+^ T cell infiltration into the vascular wall, leading to helper T cell–driven inflammatory responses within plaques. The contribution of CD8^+^ T cells is less consistently described, although some evidence suggests that cytotoxic T cells may sustain vascular injury in more advanced lesions.

The protective role of CTLA-4 against atherosclerosis has been investigated in a study on transgenic apolipoprotein E-deficient (*Apoe*^−^/^−^) mice with constitutive cell surface and intracellular expression of CTLA-4 in T cells. In this study, CTLA-4 overexpression significantly reduced atherosclerotic lesion formation and intraplaque accumulation of macrophages and CD4^+^ T cells in the aortic root compared with controls [21]. Consistent with this observation, in low-density lipoprotein receptor-deficient (*Ldlr*^−^/^−^) mice receiving a hypercholesterolemic diet, administration of anti-CTLA-4 accelerated atherosclerosis progression by inducing predominantly T cell-driven inflammation and promoting the formation of plaques with larger necrotic cores and reduced collagen content [22].

Similarly, another preclinical study on hypercholesterolemic ApoE3*Leiden mice showed that administration of anti-CTLA-4 increased vascular lesion size, whereas abatacept, a soluble CTLA-4Ig fusion protein, reduced atherosclerosis development [23].

The PD-1/PD-L1 pathway plays a protective role against atherogenesis, as highlighted in several preclinical studies. Specifically, in a study on *Ldlr*^−^/^−^ mice, PD-L1/2 deficiency led to a significantly increased atherosclerotic burden throughout the aorta and an increased number of lesional T cells. Furthermore, PD-L1/2-deficient mice exhibited higher serum levels of TNF-α and more effective antigen-presenting cells (APCs) in activating CD4^+^ T cells in vitro compared with controls [24]. In another study, administration of an anti-PD-1 antibody increased lesional inflammation in hypercholesterolemic *Ldlr*^−^/^−^ mice, with more lesional T cells and more activated T cells in para-aortic lymph nodes. Conversely, activation of the PD-1/PD-L1 pathway through administration of an agonistic PD-L1 antibody in *Ldlr*^−^/^−^ mice resulted in reduced atherosclerosis development [25].

The protective role of PD-1/PD-L1 has also been investigated in patients with coronary artery disease (CAD). A study on 76 patients with CAD and 25 healthy volunteers reported that PD-1 and PD-L1 expression was significantly downregulated on peripheral blood T cells and dendritic cells (DCs) in CAD patients compared with healthy individuals. Moreover, decreased PD-L1 expression on DCs was associated with increased T cell immune responses in CAD patients. Additionally, in vitro stimulation of PD-L1 expression attenuated the stimulatory ability of DCs on allogeneic T cell proliferation and cytokine production, including IFN-γ and IL-2 [26]. Consistently, in a study on 59 patients with CAD and 11 healthy volunteers, patients with acute coronary syndrome exhibited significantly reduced PD-L1 expression on peripheral blood Tregs compared with patients with chronic CAD and the control group [27].

LAG-3 also appears to play a regulatory role in atherosclerosis development. In preclinical models, LAG-3 deficiency or treatment with blocking anti-LAG-3 monoclonal antibodies promoted T cell activation and accumulation in plaques while not affecting plaque burden [28]. Interestingly, combination therapy with anti-PD-1 and anti-LAG-3 had an additive effect on T cell activation and cytokine production and promoted plaque infiltration by T cells. Emerging clinical data are consistent with preclinical studies. A study on 49 patients with CAD and 17 healthy volunteers showed that soluble LAG-3 levels and LAG-3 gene expression in peripheral whole blood were significantly lower in CAD patients compared with controls [29].

Overall, available evidence suggests that inhibitory immune checkpoints play a crucial role in reducing inflammation within the plaque microenvironment. Conversely, their inhibition by ICIs may be associated with atherosclerosis progression. Two primary mechanisms have been proposed for ICI-induced atherosclerosis progression: epitope sharing, i.e., activation of T cell clones reactive to autoantigens specific to atherosclerosis [30], and cytokine dysregulation, i.e., excessive production of pro-inflammatory cytokines such as TNF-α and IFN-γ [31].

In addition to autoantigens, a hypothetical mechanism is that tumor antigens may share structural similarities with vascular self-antigens, potentially promoting cross-reactivity of T cells activated by ICIs. While Chowdhury et al. [30] demonstrated that human coronary plaque T cells can cross-react with viral and self-antigens, direct preclinical or patient-derived evidence of tumor-endothelial molecular mimicry is currently lacking. This remains a speculative but relevant field for future research, as identifying such epitopes could enable longitudinal monitoring of T cell responses in patients and offer insights for atherosclerosis biology in general.

**Figure 1:**
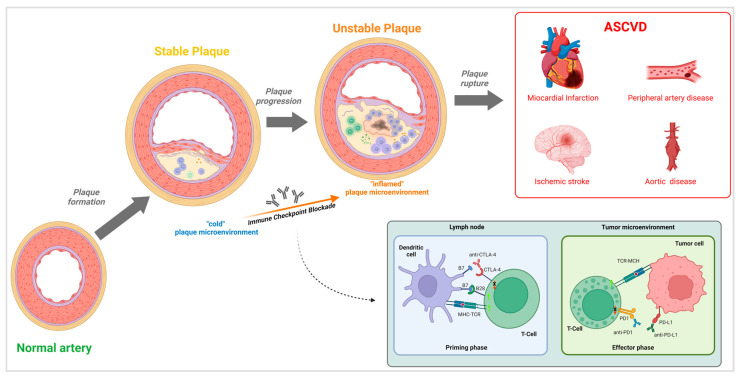
Immune checkpoint inhibitor (ICI)-induced atherosclerosis development. Cytokine dysregulation and epitope sharing promote the transition from a “cold” to an “inflamed” plaque microenvironment, with infiltration of CD4^+^ T cells and macrophages (and occasional CD8^+^ T cells in advanced lesions). This inflammatory state accelerates plaque progression, necrotic core formation, and rupture risk. The checkpoint blockade box illustrates how inhibition of CTLA-4, PD-1/PD-L1, and LAG-3 pathways removes protective signals against vascular inflammation. ASCVD = atherosclerotic cardiovascular disease, including myocardial infarction, ischemic stroke, peripheral artery disease, and aortic disease.

These mechanisms drive a shift in the plaque microenvironment from “cold” to “inflamed.” An inflamed plaque microenvironment promotes several processes that drive atherosclerosis progression, including smooth muscle cell hyperplasia, collagen deposition, macrophage activation with increased phagocytosis of LDL cholesterol, macrophage transformation into foam cells, structural plaque alterations potentially leading to necrotic core formation, and, ultimately, plaque instability with an increased risk of rupture [20] (Figure 1). Clinical–mechanistic integration. Preclinical evidence that ICIs restrain intimal inflammation (CTLA-4, PD-1/PD-L1, LAG-3) provides a biologic plausibility for clinical associations observed in retrospective cohorts and imaging sub studies (e.g., increased plaque progression or arterial inflammation). However, these data remain largely observational and heterogeneous, and causality cannot be inferred.

## 3. Long-Term Atherosclerosis-Related Cardiovascular Toxicity of Immunotherapy

To avoid ambiguity, in this review we use the following operational definitions: acute/early cardiovascular irAEs, 0–3 months after ICI initiation; delayed, 3–12 months; and long-term, >12 months after initiation (including events that arise after treatment discontinuation). Our focus here is on long-term, atherosclerosis-related events (myocardial infarction (MI), ischemic stroke, peripheral artery disease, aortic disease).

Initially, atherosclerosis-related cardiovascular toxicity was underreported in clinical trials, as it was not specifically recognized as an irAE. Most of the available evidence on atherosclerosis-related cardiovascular events derives from patients treated with anti-PD-1 or anti-PD-L1 monotherapy, reflecting their widespread use across tumor types. A smaller number of studies included patients receiving anti-CTLA-4 monoclonal antibodies, either alone or in combination with anti-PD-1 and suggest that combination regimens may be associated with accelerated progression of non-calcified plaques and higher inflammatory activity [32,33]. However, data remain limited and heterogeneous, and direct comparisons between different ICIs are lacking.

Only sporadic cases were documented, such as MI in patients enrolled in trials with atezolizumab for urothelial carcinoma [34] and pembrolizumab for Non-Small Cell Lung Cancer (NSCLC) [35]. Accordingly, in a meta-analysis assessing cardiovascular irAEs in cancer patients receiving ICIs, the reported incidence of MI was very low (0.4%; 95% CI, 0–0.07%). However, this estimate is likely conservative, since the 26 included trials were not specifically designed to evaluate cardiovascular toxicity, and only 6 of them reported MI as an irAE [36]. Likewise, another meta-analysis involving more than 20,000 patients across 68 studies reported a similarly low incidence of arterial thromboembolic events (1.1%; 95% CI, 0.5–2.1%) [35,36,37]. Tumor type itself may also influence the likelihood of acute vascular events. Lung cancer patients treated with ICIs seem to have a higher incidence of these events. Indeed, a meta-analysis of 22 NSCLC trials reported an incidence of 1.0% for MI (95% CI, 0–3.8%) and 2.0% for stroke (95% CI, 0–13.0%) [36,37,38].

There is now consistent evidence that the risk of atherosclerotic cardiovascular disease (ASCVD) is increased in patients receiving ICIs, although incidence rates reported across studies vary widely. Such variability likely reflects heterogeneity in study designs, differences in follow-up duration, and variations in patient populations with respect to primary tumor site and baseline cardiovascular risk factors. The main evidence on ASCVD in patients treated with ICIs is summarized in Table 1.

A retrospective study of 672 ICI-treated patients reported incidences of 2.2% for acute coronary syndrome and 1.6% for stroke over a median follow-up of 13 months [39]. In a large single-center registry including 3326 cancer patients treated with ICIs, Oren et al. reported that 7% of patients experienced either MI or stroke within 16 months [40].

A large matched-cohort retrospective study by Drobni et al. analyzed 2462 cancer patients treated with ICIs at Massachusetts General Hospital between 2008 and 2012, and a control cohort of 2842 cancer patients matched for age, history of cardiovascular events, and primary tumor type, who did not receive ICIs. Patients receiving ICIs showed a markedly higher risk of MI (HR 7.2; 95% CI, 4.5–11.5; *p* < 0.001), coronary revascularization (HR 3.0; 95% CI, 1.9–4.8; *p* < 0.001), and ischemic stroke (HR 4.6; 95% CI, 2.9–7.2; *p* < 0.001) [32]. Moreover, a case-crossover analysis within the ICI cohort demonstrated an increased incidence of acute vascular events in the two years following treatment compared with the two years prior: 4.2% vs. 2.32% (HR 4.78; 95% CI, 3.50–6.53; *p* < 0.001). Finally, an imaging sub study in 40 melanoma patients treated with ICIs revealed more than a threefold increase in the rate of atherosclerotic plaque progression after therapy initiation (from 2.1% annually before ICIs to 6.7% annually thereafter) [41].

Similarly, a case–control study in lung cancer patients [42] reported accelerated progression of aortic atherosclerotic plaque among those treated with ICIs. Specifically, the annual progression rate of non-calcified plaque volume was sevenfold higher in the ICI group than in controls (11.2% vs. 1.6% per year; *p* = 0.001), suggesting that ICIs may foster the development of unstable plaques prone to rupture. The progression of non-calcified plaques was more pronounced in patients receiving combination ICI therapy compared with single-agent therapy. Consistent findings emerged from a recent Chinese study, in which coronary artery calcium (CAC) volume assessed on routine thoracic computed tomography (CT) scans increased in advanced NSCLC patients after ICI initiation, in parallel with the occurrence of cardiovascular events, indicating that calcified plaques also tend to progress [42].

Beyond promoting an increase in plaque volume, ICIs have also been implicated in exacerbating plaque inflammation, a process that may contribute to plaque instability and a higher risk of acute vascular events. In support of this, a retrospective analysis of 20 melanoma patients treated with ICIs evaluated atherosclerotic inflammatory activity using serial fluoro-D-glucose positron emission tomography (18F-FDG PET)/CT scans performed before and during treatment. Following ICI initiation, non-calcified and mildly calcified segments showed a significant rise in FDG uptake, consistent with enhanced inflammatory activity in early-stage lesions. These findings suggest that ICIs may affect local innate immune cells and amplify inflammation, particularly in early non-calcified or mildly calcified plaques [43]. Further studies confirmed that ICIs can promote vascular inflammation even in lung cancer patients without pre-existing arterial inflammation [33].

The duration of ICI exposure also appears to influence cardiovascular risk. A recent study reported that the risk of atherothrombotic events (ATEs) was approximately doubled in patients receiving ICIs compared with controls. While the cumulative risk of ATEs increased by 41% at one year in the ICI group, no significant rise was observed in patients exposed to ICIs for less than one year [44]. Importantly, as with other immune-related adverse events, the heightened cardiovascular risk may persist even after discontinuation of ICIs. Indeed, a recent report demonstrated that among ICIs recipients, a prior history of ASCVD was strongly associated with recurrent ASCVD events, which could occur even after treatment cessation [45].

Overall, the available data support the hypothesis of a link between ICIs and accelerated atherosclerosis, but causality remains unproven. Importantly, these findings should be framed as an emerging management challenge rather than a limitation of immunotherapy, whose survival benefits remain substantial [46,47,48,49,50].

**Table 1:** Summary of clinical evidence on ASCVD in patients treated with IT.

Author, Year	Population	Study Design	Main Observation	Relevance (H/M/L) and Limitation
Drobni, 2020 [41]	2842 pts and 2842 controls	Retrospective, paired cohorts	- ICI-treated patients showed higher risk of AMI, stroke and coronary revascularization. - Increased risk in two years after ICI start vs. 2 years before (HR 4.78).	**M** Retrospective but paired controls
Drobni, 2020 [41]	40 ICI-treated melanoma patients	Retrospective Sub analysis	- Increased rate of yearly plaque progression (2.1% pre vs. 6.7% post-ICI).	**L** Retrospective, small sample size
Gong, 2024 [33]	487 ICI-treated NSCLC pts; 971 controls	Retrospective, case–control	- Atherosclerosis-related events 4.9% in ICI-treated vs. 1.4% in controls.	**M/L** Retrospective, adequate sample size
Nso, 2020 [36]	4622 pts receiving ICIs	Meta-analysis	- AMI incidence: 0.4%	**M** Meta-analysis, possible reporting bias
Solinas, 2020 [37]	20,273 pts	Meta-analysis	- Arterial thromboembolic events incidence 1.1% - Stroke rate 1.1% - AMI rate 0.7%	**M** Retrospective; few trials with control arm, based on initial clinical experience with IT
Hu, 2017 [38]	4828 NSCLC pts	Meta-analysis	- AMI incidence 1% - Stroke incidence 2% - Heart failure incidence 2%	**M/L** Retrospective, post hoc analysis, data on old trials
Oren, 2020 [40]	3326 pts receiving ICIs	Retrospective	- Incidence of cardiovascular toxicity: 2.4% - AMI rate 7% - Stroke rate 7% - Heart failure 11%	**L** Retrospective, single center, short follow-up
Drobni, 2023 [32]	40 ICI-treated NSCLC patients paired with 20 controls	Retrospective, case–control	- Yearly rate of plaque progression 10.1% with ICIs vs. 5.95% in controls (p = 0.025). - Yearly rate of calcified plaque progression 11.2% with ICIs vs. 1.6% in controls (p = 0.001).	**L** Retrospective, small sample size
Tan, 2024 [45]	366 pts no control arm	Cohort study	7.1% incidence of ASCVD events (median follow-up of 3.4 years) which could occur even after treatment stop. Previous ASCVD predicted toxicity.	**L** Retrospective, single center, small sample size
Nichetti, 2019 [46]	217 ICI-treated NSCLC pts	Retrospective	- Incidence of arterial thromboembolic events: 6.5% (AMI: 1%; stroke: 4.1%).	**L** Retrospective, small sample size
Dolladille, 2021 [47]	48 RCTs (29,592 pts) with control arm	Meta-analysis	Increased risk of AMI (7.4/1000 pts on ICIs, OR 1.51 vs. controls) and cerebral arterial ischaemia (8.8/1000 pts, OR 1.56 vs. controls).	**H/M** Retrospective, post hoc analysis limited to RCTs reporting CV events
Salem, 2018 [48]	31,321 adverse events reports (on a total of 16,343.451)	Retrospective based on pharmacovigilance reports	- AMI incidence 0.53% - Stroke incidence 0.62%	**M** Retrospective; potential reporting bias, based on initial clinical experience with IT
Wang, 2022 [50]	289 ICI-treated melanoma vs. 357 controls	Retrospective	- ICI treatment was associated with an increased risk of MACE (*p* = 0.03) especially in patients with a history of MACE.	**L** Retrospective, relatively small sample size

**High-H** (large size, prospective, control arm, possibly RCTs), **medium**-**M** (pharmacovigilance or similar with potential reporting bias but large sample size, meta-analysis), **low-L** (small sample size, old data, single center). RCTs: randomized controlled trials; ASCVD: atherosclerotic cardiovascular disease; NSCLC: non-small cell lung cancer; IT: immunotherapy; ICIs: immune checkpoint inhibitors; AMI: acute myocardial infarction.

This risk increases over time to a potential competitive cause of death in patients who benefit from long-term disease control by IT [51]. For these reasons, cardiovascular risk assessment (both baseline and treatment-related) should be a priority in patients treated with long-term ICI. Therefore, considering the potential worsening of cardiovascular risk due to long-term IT, treating patients who already have a high risk or who are already suffering from heart disease is particularly challenging. Cardiological/cardioncological specialist management is therefore recommended in this category of patients, aimed at setting up a specific program for cardiovascular prevention and monitoring.

Cardiovascular risk assessment in patients treated with long-term ICIs should be a priority for oncologists, who are often mainly focused on disease control/cure. Patients with high cardiovascular risk or a history of cardiovascular disease need special management by cardiologist or cardio-oncologist to establish a cardiovascular prevention and surveillance program [16,52].

## 4. Cardiovascular Risk Assessment and Monitoring for ASCVD

The survival benefits achieved with ICIs, coupled with the risk of delayed or long-term toxicities, confront clinicians with unprecedented challenges in the field of cardiovascular health. Importantly, these challenges should be viewed from a balanced perspective: while immunotherapy has transformed survival expectations across cancer types, cardiovascular toxicity represents an emerging management issue rather than a limiting factor of therapy. Consequently, the stratification of cardiovascular risk in patients receiving ICIs has become a critical issue. Two main determinants contribute to cardiovascular toxicity risk: the first, predating cancer diagnosis, is the patient’s baseline cardiovascular risk profile, which has historically been underestimated in oncology; the second is the oncologic treatment itself, traditionally regarded as the primary source of risk. To date, available tools are imperfect: while they highlight associations between ICIs and cardiovascular risk, they are not yet validated to establish direct causality. Therefore, their integration into clinical decision-making should remain cautious and complementary to established cardiovascular prevention strategies. Both factors should be carefully evaluated to obtain a comprehensive estimate of cardiovascular risk.

For baseline cardiovascular risk assessment, current tools such as SCORE2 may underestimate event probability, as they do not account for the additional risk conferred by cancer itself, which should therefore be considered an independent contributing factor [53]. Regarding treatment-related cardiovascular toxicity, the European Society of Cardiology (ESC) guidelines recommend the use of the HFA-ICOS classification system. However, this tool has not yet undergone full validation and does not provide specific guidance on the long-term cardiovascular effects of ICIs [54].

In patients scheduled for long-term treatment with ICIs, the ESC guidelines recommend a baseline cardiovascular assessment, along with periodic monitoring of ECG, echocardiography, troponin, and brain natriuretic peptide (BNP) levels. However, these monitoring strategies may fail to detect potential progression of atherosclerotic plaque. To date, no established surveillance program exists for the early diagnosis of ASCVD in patients receiving ICIs. The previously cited evidence showing the potential to detect plaque enlargement by CT scans and/or increased glucose uptake by PET/CT examinations that patients routinely undergo for tumor assessment during and after ICI therapy highlights the importance of heightened vigilance by radiologists and nuclear medicine physicians in recognizing any changes in plaque characteristics. Moreover, clinicians should maintain a high level of vigilance for any early signs of ASCVD in order to initiate an appropriate diagnostic work-up when indicated.

Finally, as part of the baseline evaluation, it is essential that oncologists assess key risk factors such as smoking, obesity, physical inactivity, diet, dyslipidemia, diabetes, and hypertension, to implement aggressive risk-reduction strategies, as outlined in the following section.

## 5. Preventive Strategies

While ICIs have extended survival across a broad spectrum of tumors, they may also lead to long-term cardiovascular complications, including the progression of atherosclerosis and subsequent ACVD. In this scenario, it is important to take a comprehensive approach to oncology patients receiving ICIs, incorporating cardiovascular risk prevention strategies, with an aggressive management of ASCVD risk factors [55]. Specifically, substantial efforts should be made to optimize the classic main modifiable cardiovascular risk factors for ASCVD, such as LDL cholesterol levels, hypertension, smoking, and diabetes mellitus (DM) (Table 2).

The first step in this approach is lifestyle modification. Smoking cessation should be recommended for all active smoker cancer patients, along with participation in a smoking cessation program that includes individual counseling, psychological support and nicotine replacement therapy, if necessary.

Clinicians should also encourage physical activity, adapting recommendations to the patient’s performance status and cancer-related limitations. When feasible, 150–300 min per week of moderate-intensity aerobic physical activity is recommended. If this target is unachievable, reducing sedentary time by incorporating light physical activity throughout the day is still beneficial.

Additionally, cancer patients should be advised to follow a healthy diet. Specifically, alcohol abstinence or reduced consumption is recommended, along with a Mediterranean diet that emphasizes plant-based foods rich in fiber and reduces the intake of saturated fats, salt, and free sugars, particularly sugar-sweetened beverages. Limiting processed meats and including fish, preferably fatty fish, at least once per week is also recommended. For cancer patients with high-volume metastatic disease connected to cachexia or those with primary tumors of the head and neck, lung, upper gastrointestinal tract, or pancreas, there is a significant risk of malnutrition. In these cases, a general recommendation for a healthy diet should be complemented by a thorough nutritional assessment to determine the need for dietary supplements aimed at preventing weight loss and malnutrition [56]. Conversely, obese cancer patients should undergo a nutritional assessment focused on weight loss, to reduce blood pressure, dyslipidemia, and risk of DM type 2, thereby improving their overall cardiovascular risk profile.

Baseline LDL cholesterol assessment before initiating ICIs should be included in the comprehensive cardiovascular risk evaluation. In cases of dyslipidemia, lipid-lowering therapy should be started. In patients without ASCVD, LDL cholesterol levels below 100 mg/dL are desirable, while in patients with known ASCVD, the target LDL cholesterol level should be below 55 mg/dL. Retrospective studies suggest that statin therapy may attenuate the association between ICI treatment and atherosclerotic plaque progression [40]. Moreover, statin use has been associated with an improved objective response to ICIs [57]. These findings support the use of statins as the first-line lipid-lowering therapy in cancer patients receiving ICIs. Recently, Proprotein Convertase Subtilisin/Kexin Type 9 (PCSK9) inhibitors have been shown to reduce LDL cholesterol levels and ASCVD risk across multiple patient cohorts. Given that PCSK9 promotes peripheral immune tolerance and inhibits cancer cell immune recognition, the use of PCSK9 inhibitors may potentially enhance the efficacy of ICIs [58]. This makes these agents a particularly promising option for cancer patients receiving ICIs.

Cancer patients receiving ICIs should undergo baseline and periodic blood pressure monitoring, as some evidence suggests that these patients may have undiagnosed hypertension [59]. In patients with newly diagnosed hypertension, antihypertensive treatment is recommended. In those with preexisting but inadequately controlled hypertension, therapy should be adjusted according to hypertension treatment guidelines, considering the patient’s age and risk category. ACE inhibitors (ACEi) or angiotensin II receptor blockers (ARBs) should be preferred as first-line therapy. The addition of a beta-blocker may be considered, as some retrospective studies have reported a weak favorable association between beta-blocker use and response to ICIs (not always found) [60,61,62]. In addition, preclinical and translational studies have shown that T lymphocytes express β-adrenergic receptors, and that β-adrenergic blockade may enhance T cell function by reducing adrenergic immunosuppression [63]. This immunomodulatory effect provides a potential mechanistic rationale for the observed association between beta-blocker use and improved outcomes in ICI-treated patients [60].

Finally, cancer patients should be evaluated for fasting blood glucose levels, and HbA1c should be assessed if diabetes is suspected. In patients with a confirmed diabetes diagnosis, metformin is the first-line therapy. Retrospective studies suggest that metformin use may be associated with an improved response to ICI treatment [64,65] but this evidence is far from certainty [66]. In patients with type 2 diabetes and chronic kidney disease (CKD) or known ASCVD, the use of a Sodium-glucose co-transporter 2 (SGLT2) inhibitor is recommended, and their role is expanding beyond traditional indications, with emerging evidence also supporting a potential benefit in the cardio-oncology setting [67,68]. In diabetic patients receiving ICIs, glucagon-like peptide-1 receptor agonists (GLP-1 RAs) may also represent a valuable option, as they can reduce atherosclerosis through both direct and indirect mechanisms [69]. GLP-1 RAs act directly on endothelial cells by decreasing cholesterol accumulation, vascular inflammation, and vascular smooth muscle activation and proliferation. Indirectly, they contribute to atherosclerosis reduction by lowering blood pressure, body weight, and glycated hemoglobin, all of which play a key role in the pathogenesis of atherosclerosis.

The potential association between ICI efficacy and lipid-lowering or anti-diabetic therapy, as suggested by retrospective studies, requires confirmation through prospective trials. Additionally, prospective studies are needed to establish the optimal strategy for preventing atherosclerosis progression in cancer patients receiving ICIs.

From a clinical perspective, the evidence summarized above suggests that cardio-oncology care pathways should integrate three key steps: (1) baseline cardiovascular risk assessment prior to ICI initiation, including evaluation of traditional risk factors and previous ASCVD; (2) structured monitoring during treatment, using biomarkers (troponin, BNP) and imaging (echocardiography, CT/PET when available); and (3) aggressive preventive interventions, with both lifestyle modifications and pharmacological therapies (statins, PCSK9 inhibitors, SGLT2 inhibitors, GLP-1 receptor agonists) tailored to the patient profile. Embedding these measures into a multidisciplinary care framework involving oncologists, cardio-oncologists, and other specialists (nutritionists, endocrinologists, physiotherapists) may help preserve the survival benefits of ICIs while minimizing long-term cardiovascular risks.

## 6. Future Research Perspectives

The increased risk of ASCVD in cancer patients receiving long-term IT should not be considered as an “all or nothing phenomenon” purely. Apart from the role of drugs themselves (ICI combinations seemed to be associated with higher risk), individual determinants of such risk are unknown to date. Single patient T lymphocytes could be more prone to overreact to IT stimuli leading to accelerated atherosclerosis process. While this increased immune-cells sensitivity to IT is based on genetic alterations or mediated by the presence of cancer itself has not been investigated and represents a field of interest for future research. The identification of specific “pro-atherogenic” predictive factors could allow a more careful patients selection and a tailoring of cardiovascular preventive strategies and follow-up.

The ability to recognize subclinical changes in the vascular wall in the initial phase of the IT-induced atherosclerotic process is another issue worthy of dedicated research. PET scans, widely used in cancer diagnosis and re-evaluation during treatment, are not the ideal candidates. The evidence of increased glucose uptake is usually limited to great vessels while most ASCVD events are related to medium-to-small vessels (i.e., coronary arteries). Research should so be focused on the use of already available cancer imaging to identify early imaging markers of the pro-atherogenic effect of IT (i.e., by means of dedicated software).

## 7. Conclusions

While long-term ICI exposure is increasingly associated with ASCVD events, these findings should not overshadow the transformative survival benefits that immunotherapy has provided across multiple cancers. Rather than representing a limitation, cardiovascular risk should be regarded as an emerging management challenge that requires proactive surveillance and prevention. Future cardio-oncology research should aim at integrating cardiovascular risk assessment and management into immunotherapy care pathways, ensuring that survival gains are fully preserved and enhanced by comprehensive patient care. Advanced patients experiencing long-term disease control with IT as well as early-disease patients receiving adjuvant or neo-adjuvant ICI treatments should enter a dedicated cardiovascular care path focusing on the identification of classic CV risk factors coupled with their aggressive management.

## Figures and Tables

**Table 2:** Proposed preventive strategies for cardiovascular risk factors control in cancer patients receiving long-term ICIs.

Risk Factor	Goal	Strategy
Smoking	Complete smoking cessation	Smoking cessation program with individual counseling. Psychological support. Nicotine replacement (if necessary).
Obesity and physical inactivity	Encourage physical activity (adapt recommendations to patient’s performance and cancer-related limitations)	150–300 min per week of moderate-intensity aerobic physical. Reducing sedentary time. Incorporate light physical activity throughout the day.
Diet	Switch to Mediterranean diet	Alcohol abstinence or reduced consumption. Plant-based foods rich in fiber. Reduce saturated fats, salt, and free sugars (particularly sugar-sweetened beverages). Limit processed meats. Include fish (preferably fatty fish) at least once per week.
Cholesterol	Lower LDL cholesterol levels (below 100 mg/dL if no ASCVD and below 55 mg/dL if ASCVD)	Heathy diet Lipid-lowering therapy (statins, PCSK9)
Diabetes	Optimal blood glucose control	Metformin as the first-line therapy. If type 2 diabetes and CKD or known ASCVD, use an SGLT2 inhibitor. GLP-1 receptor agonists may also represent a valuable option.
Hypertension	Lower blood pressure levels	Periodic blood pressure measurement. If new hypertension, antihypertensive treatment is recommended If uncontrolled hypertension, adjust therapy with ACEi or ARBs as preferred first-line therapy (addition of beta-blockers may be considered).

ASCVD: atherosclerotic cardiovascular disease; PCSK9: Proprotein Convertase Subtilisin/Kexin Type 9; CKD: chronic kidney disease; SGLT2: Sodium-glucose co-transporter 2; GLP-1: glucagon-like peptide-1; ACEi: angiotensin-converting enzyme inhibitors; ARBs: angiotensin II receptor blockers.
